# *ROR2* expression predicts human induced pluripotent stem cell differentiation into neural stem/progenitor cells and GABAergic neurons

**DOI:** 10.1038/s41598-023-51082-4

**Published:** 2024-01-06

**Authors:** Takuya Kuroda, Satoshi Yasuda, Satoko Matsuyama, Takumi Miura, Rumi Sawada, Akifumi Matsuyama, Yumiko Yamamoto, Masaki Suimye Morioka, Hideya Kawaji, Takeya Kasukawa, Masayoshi Itoh, Hidenori Akutsu, Jun Kawai, Yoji Sato

**Affiliations:** 1 Division of Cell-Based Therapeutic Products, National Institute of Health Sciences, Kawasaki, Kanagawa Japan; 2 Life Science Technology Project, Kanagawa Institute of Industrial Science and Technology, Kawasaki, Kanagawa Japan; 3 Department of Quality Assurance Science for Pharmaceuticals, Graduate School of Pharmaceutical Sciences, Nagoya City University, Nagoya, Aichi Japan; 4 Center for Reverse TR, Osaka Habikino Medical Center, Osaka Prefectural Hospital Organization, Habikino, Osaka Japan; 5 Center for Regenerative Medicine, National Center for Child Health and Development, Setagaya, Tokyo Japan; 6 RIKEN Center for Integrative Medical Sciences, Yokohama, Kanagawa Japan; 7 Research Center for Genome and Medical Sciences, Tokyo Metropolitan Institute of Medical Science, Setagaya, Tokyo Japan; 8 Division of Drugs, National Institute of Health Sciences, 3-25-26 Tonomachi, Kawasaki-ku, Kawasaki, Kanagawa 210-9501 Japan; 9 Department of Cellular and Gene Therapy Products, Graduate School of Pharmaceutical Sciences, Osaka University, Suita, Osaka Japan

**Keywords:** Pluripotent stem cells, Stem-cell differentiation

## Abstract

Despite the development of various in vitro differentiation protocols for the efficient derivation of specific cell types, human induced pluripotent stem cell (hiPSC) lines have varing ability to differentiate into specific lineages. Therefore, surrogate markers for accurately predicting the differentiation propensity of hiPSC lines may facilitate cell-based therapeutic product development and manufacture. We attempted to identify marker genes that could predict the differentiation propensity of hiPSCs into neural stem/progenitor cells (NS/PCs). Using Spearman’s rank correlation coefficients, we investigated genes in the undifferentiated state, the expression levels of which were significantly correlated with the neuronal differentiation propensity of several hiPSC lines. Among genes significantly correlated with NS/PC differentiation (*P* < 0.01), we identified *ROR2* as a novel predictive marker. *ROR2* expression in hiPSCs was negatively correlated with NS/PC differentiation tendency, regardless of the differentiation method, whereas its knockdown enhanced differentiation. *ROR2* regulates NS/PC differentiation, suggesting that *ROR2* is functionally essential for NS/PC differentiation. Selecting cell lines with relatively low *ROR2* expression facilitated identification of hiPSCs that can differentiate into NS/PCs. Cells with *ROR2* knockdown showed increased efficiency of differentiation into forebrain GABAergic neurons compared to controls. These findings suggest that *ROR2* is a surrogate marker for selecting hiPSC lines appropriate for NS/PC and GABAergic neuronal differentiations.

## Introduction

Human pluripotent stem cells (hPSCs) can differentiate in vitro to form all embryonic germ layers, including the ectoderm, and self-renew in vitro^1^. This may allow for new applications of hPSC in regenerative medicine and cell therapy. There have been numerous attempts to introduce human induced pluripotent stem cells (hiPSCs) to patients, and some of them are being tested in clinical trials^2,3^. In addition, numerous clinical-grade human embryonic stem cell and hiPSC lines have recently been established as raw materials for cell-based therapeutic products (CTPs)^4–6^. In the case of autologous transplantation where the patient’s cells are used for treatment, multiple cell lines can be established from hiPSC colonies. However, hPSC lines have individual differentiation propensities to generate specific cell lineages in vitro^7,8^, which translates to low reproducibility and is time-consuming and costly.

Multiple factors, such as partial reprogramming, incomplete silencing of transgenes, and gene misregulation by insertional mutagenesis, may cause the variations observed in the hPSC line differentiation^9^. Even hiPSC lines established from the same cells by the same reprogramming method may have varying differentiation propensities. The reasons underlying this variation among hiPSC lines are inadequately explained and remain unclear. Therefore, selecting hPSC lines that can efficiently differentiate into target cells is crucial for CTP application and in regenerative medicine.

This necessitates a marker for predicting the differentiation propensity by measuring gene expression levels in the undifferentiated state. In our previous studies, we attempted to identify markers of pluripotency status to predict the differentiation propensity in hiPSC lines using rank correlation analysis and found *SALL3* and *CXCL4* to be markers of three-germ layer differentiation and cardiomyocyte differentiation, respectively^10,11^, suggesting that the same approach can be used to identify predictive markers of the propensity to differentiate into other lineages.

HiPSC-derived neural stem/progenitor cells (NS/PCs) can differentiate into various mature neurons, astrocytes, oligodendrocytes, and other cell types, and are an important part of hiPSC-based transplantation therapy. A clinical study of hiPSC-derived NS/PC transplantation for subacute spinal cord injury is underway in Japan^12^. Here, we attempted to identify genes for potentially predicting the differentiation propensity of hiPSCs into NS/PCs, the stem/progenitor cells of various mature nerves. Using 10 hiPSC lines, we investigated genes that were significantly correlated with the tendency of NS/PC differentiation.

## Results

### Evaluation of differentiation potential of hiPSCs using two types of NS/PC differentiation methods

**Figure 1:**
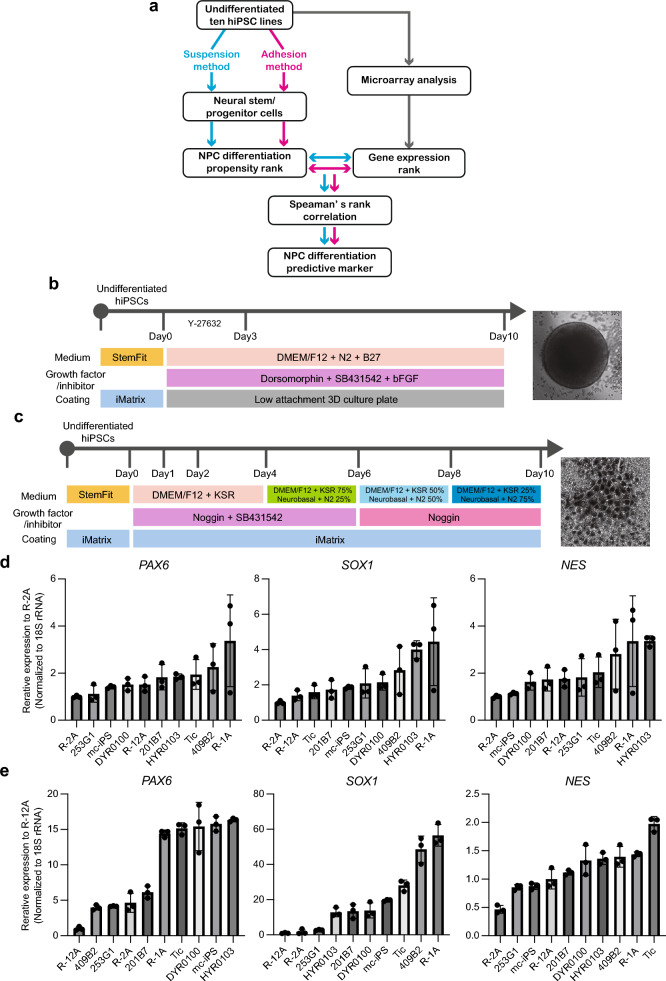
Profiles of hiPSC lines showing NS/PC differentiation propensities. (**a**) Outline of workflow for identifying biomarkers for predicting the NS/PC differentiation propensity of hiPSCs. (**b**) Schematic of culture procedures for NS/PC differentiation in suspension culture. (**c**) Schematic of culture procedures for NS/PC differentiation in adhesion culture. (**d**) qRT-PCR analysis of the mRNA levels of the NS/PC markers *PAX6*, *SOX1,* and *NES*. Total RNA was isolated from NS/PCs derived from 10 hiPSC lines differentiated using the suspension method. (n = 3, biological replicates) Using one-way ANOVA, significant differences in *PAX6* (*P* < 0.05), *SOX1* (*P* < 0.01), and *NES* (*P* < 0.05) were observed among cell lines. (**e**) qRT-PCR analysis of the mRNA levels of the NS/PC markers *PAX6*, *SOX1,* and *NES*. Total RNA was isolated from NS/PCs derived from 10 hiPSC lines differentiated using the adhesion method. (n = 3, biological replicates) Using one-way ANOVA, significant differences in *PAX6* (*P* < 0.0001), *SOX1* (*P* < 0.0001), and *NES* (*P* < 0.0001) were observed among cell lines.

We hypothesized that certain significant attributes of hiPSCs determine their propensity to differentiate into specific cells and attempted to identify potential marker genes whose expression in hiPSCs is significantly correlated with the tendency of differentiation into NS/PCs. The approach toward identifying differentiation propensity markers was essentially based on a statistical comparison of the gene expression profiles of undifferentiated hiPSCs and the in vitro NS/PC differentiation propensity of each cell line using the rank correlation method, as described in our previous studies^10,11^ (Fig. 1a). We first obtained the gene expression profiles of 10 hiPSC lines (Supplementary Table 1) using microarray analysis published in our previous study^10^. The defined filtering criteria were used to identify 3,362 probes with significantly different expression levels among the hiPSC lines (GSE88963). Subsequently, to examine the differences in neural differentiation propensity among the hiPSC lines, they were differentiated into NS/PCs using two differentiation methods, suspension and adhesion culture, to obtain two independent and significantly correlated groups of genes. We hypothesized that the genes whose expression levels correlated with neuronal differentiation potential in common when differentiated by the two methods were likely to be essential for neuronal differentiation. We employed different culture methods (suspension or adhesion) with different medium compositions, considering that genes showing significant correlations in common, even in completely independent cell-differentiation methods, are more important factors. For suspension culture differentiation, we used a BMP4 inhibiting method based on protocols in previous reports, with certain modifications^13^ (Fig. 1b). Adhesion culture differentiation was induced through the dual inhibition of SMAD signaling, as described in a previous study^14^ (Fig. 1c). Using total RNA isolated from NS/PC after 10 days of differentiation, the expression levels of three NS/PC marker genes (*PAX6*, *SOX1*, and *NES*) were examined using quantitative reverse transcription-PCR (qRT-PCR). In the suspension method, differences in the expression levels of *PAX6* and *SOX1* between NS/PCs from different hiPSC lines were within fivefold, whereas in the adhesion method, these differences were more than 15-fold, suggesting that the adhesion method more significantly induces cells with the NS/PC differentiation potential into NS/PCs than the suspension method (Fig. 1d,e).

### Screening of NS/PC differentiation marker genes

**Figure 2:**
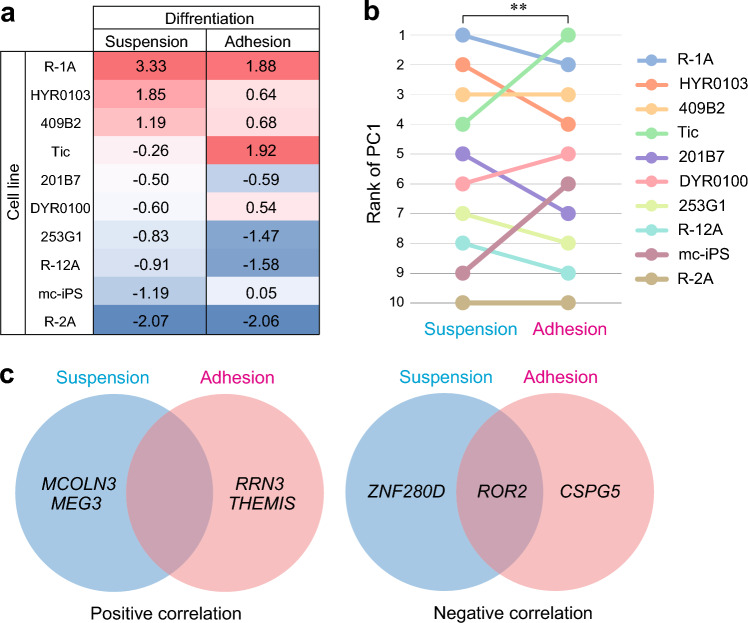
Identification of predictive markers of NS/PC differentiation using rank correlation analysis. (**a**) Expression profiles for NS/PC marker genes were summarized using PCA. The number indicates the first principal component score (PC1) of each NS/PC differentiation method among the 10 hiPSC lines. (**b**) The line graph represents the rank of the PC1 score of each NS/PC differentiation method among the 10 hiPSC lines. **P < 0.01, Spearman’s rank correlation coefficients. (**c**) Venn diagrams illustrate identified NS/PC differentiation propensity markers that indicated statistically significant correlations (*P* < 0.01, Spearman’s rank correlation coefficients). Positively correlated genes (left) and negatively correlated genes (right) were identified as candidate marker genes with a common correlation between the two differentiation methods. All candidate genes of NS/PC differentiation propensity markers are listed in Table 1.

To reduce the variables indicating the differentiation propensity of each cell line, NS/PC marker gene expression data were analyzed using principal component analysis (PCA), and the first principal component score (PC1) for NS/PC differentiation was calculated. Because it generates principal components in order starting with PC1, to consider as much information on each variable as possible, we used PC1 as an indicator of each cell line's propensity for NS/PC differentiation based on the expression levels of multiple marker genes. The explained variance of PC1 was 88.16% for the suspension method and 65.17% for the adhesion method. Based on the PC1 results, we ranked the 10 hiPSC lines in descending order of PC1 for NS/PCs (Fig. 2a,b); R-2A cells had the lowest efficiency for NS/PC differentiation regardless of the differentiation method. We observed a significant positive rank correlation between the two differentiation methods (*r*_*s*_ = 0.818, *P* < 0.01). Subsequently, Spearman’s rank correlation coefficients between the gene expression (microarray data) and NS/PC differentiation ranks (PC1) were determined. We identified differentiation propensity marker candidates with statistically significant correlations (*P* < 0.01). The Spearman’s correlation coefficient describes positive and negative correlations. There were two positively correlated and two negatively correlated genes each in both suspension and adherent cultures (Table 1). Among the identified potential marker genes, *ROR2* was the only gene showing a common correlation between the two differentiation methods (Fig. 2c).

**Table 1:** Potential marker genes showing statistically significant correlations.

Adhesion method				Suspension method			
Positive		Negative		Positive		Negative	
Gene	*r*_s_	Gene	*r*_s_	Gene	*r*_s_	Gene	*r*_s_
*THEMIS*	0.867	*CSPG5*	− 0.903	*MCOLN3*	0.830	*ROR2*	− 0.842
*RRN3*	0.830	*ROR2*	− 0.891	*MEG3*	0.794	*ZNF280D*	− 0.818

### *ROR2* is a marker for NS/PC differentiation propensity of hiPSCs

**Figure 3:**
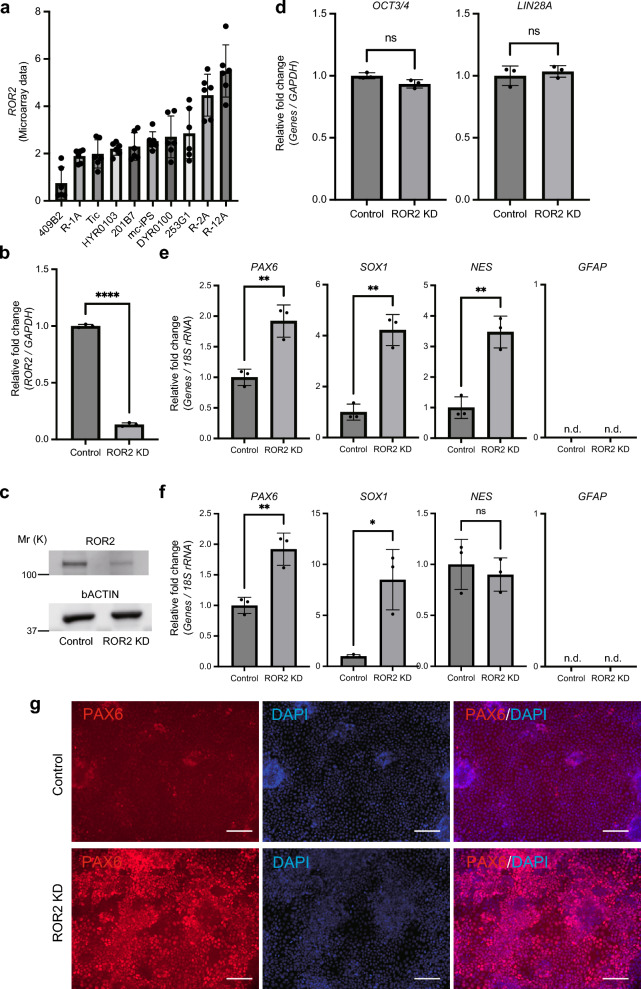
*ROR2* negatively regulates the differentiation of hiPSCs into NS/PCs. (**a**) Microarray data of *ROR2* expression in 10 hiPSC lines (n = 6, biological replicates). Using one-way ANOVA, a significant difference in *ROR2* expression was observed between cell lines (*P* < 0.0001). (**b**) *ROR2* KD was confirmed using qRT-PCR analysis (n = 3, biological replicates). (**c**) Western blotting analysis of the total extracts obtained from control and *ROR2* KD cells. β-actin was used as a loading control. Molecular weight is indicated as Mr (k). (**d**) qRT-PCR analysis of undifferentiated hPSC markers, *OCT3/4* and *LIN28A*. Total RNA was isolated from R-2A *ROR2* KD cells and R-2A control shRNA cells in the undifferentiated state (n = 3, biological replicates). (**e**, **f**) qRT-PCR analysis of NS/PC and astrocyte marker genes in NS/PCs derived from *ROR2* KD and control shRNA cells (n = 3, biological replicates). Suspension method (**e**) and adhesion method (**f**) are shown. (**g**) Immunofluorescence staining of PAX6 (red) and DAPI (blue) in control (upper) and *ROR2* KD (lower) cells. Scale bars, 100 µm. *P < 0.05, **P < 0.01, ****P < 0.0001 (two-tailed unpaired t-test). Error bars represent mean ± SD.

*ROR2* encodes ROR2, a WNT receptor protein, which is essential for inducing hiPSC differentiation^15^. Although WNT is reported that intricately regulated in neuronal differentiation, it remains unclear whether *ROR2* regulates NS/PC differentiation in hiPSCs. To examine the role of *ROR2* in regulating the NS/PC differentiation propensity of hiPSCs, we performed loss-of-function experiments by introducing lentiviral vectors containing *ROR2* shRNA into R-2A cells that showed the lowest propensity for neuronal differentiation. There was a more than fivefold difference between the highest and lowest expression levels of *ROR2* mRNA in the 10 cell lines, with the R-2A line showing the second highest expression level (Fig. 3a). The knockdown (KD) efficiency of *ROR2* in *ROR2* KD cells was confirmed by the expression levels of *ROR2* transcripts and proteins (Fig. 3b,c). In addition, *ROR2* KD showed little effect on the mRNA expression of the pluripotency markers (*OCT3/4* and *LIN28A*) in undifferentiated hiPSCs (Fig. 3d). Furthermore, when *ROR2* KD cells were subjected to NS/PC differentiation using the two methods, NS/PCs derived from *ROR2* KD cells showed significantly higher expression levels of NS/PC marker genes (*PAX6*, *SOX1* and *NES*) than control cells, except for *NES* in the adhesion culture method (Fig. 3e,f); this lead to increases in the PC1 scores of NS/PCs derived from the suspension and adhesion cultures by 7.8 and 1.0, respectively, compared with NS/PCs from the controls. In contrast, the expression of *GFAP*, an astrocyte marker, was not observed in control cells and *ROR2* KD cells for either differentiation method, suggesting that differentiation into astrocytes did not occur at day 10. The results were reproducible even when hiPSCs with *ROR2* KD by shRNA of a different sequence were differentiated into NS/PCs, excluding the possibility of off-target effects of *ROR2* KD on differentiation (Supplementary Fig. 1). Moreover, immunofluorescence images indicated the upregulation of PAX6 protein expression in *ROR2* KD cells after inducing adhesion culture NS/PC differentiation (Fig. 3g). Furthermore, we found the overexpression of *ROR2* in 253G1 cells and a significant decrease in the expression of *PAX6* after NS/PC differentiation with the adhesion method, which was consistent with the knock-down experiment. However, the expression of *SOX1* and *NES* remained unchanged compared with that in hiPSCs transfected with the control vector, and there was no significant effect of the *ROR2* overexpression on the NS/PC differentiation propensity (Supplementary Fig. 2). Together, these results indicate that *ROR2* negatively regulates the differentiation of hiPSCs to NS/PCs.

### Microarray analysis of signaling in response to *ROR2* knockdown during NS/PC differentiation

**Figure 4:**
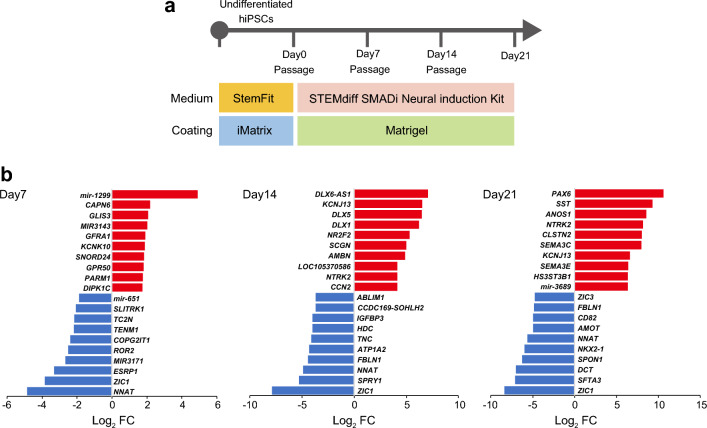
Microarray analysis of signaling in response to *ROR2* knockdown during NS/PC differentiation. (**a**) Schematic of culture procedures for NS/PC differentiation in adhesion culture. HiPSCs were differentiated into NS/PCs using the STEMdiff SMADi Neural Induction Kit (Stem Cell Technologies) monolayer culture protocol, according to the manufacturer’s instructions. (**b**) Total RNAs were obtained at three points on days 7, 14, and 21 and were analyzed using DNA microarrays (Clariom D Assay). The graphs show the fold changes of the top and bottom differentially expressed genes from a Clariom D Assay comparing the control and *ROR2* KD cells. *ROR2* KD cells showed decreases in *ROR2* expression to 18%, 16%, and 53% of that in the controls on days 7, 14, and 21, respectively, though *ROR2* was outside the top 10 down-regulated genes on days 14 and 21.

To explore the possible molecular mechanism underlying the negative effects of *ROR2* in NS/PC differentiation, we compared gene-expression profiles of *ROR2* KD cells and control cells during the differentiation of hiPSCs into NS/PCs. NS/PC differentiation of *ROR2* KD cells was performed according to another protocol using a commercially available in vitro differentiation medium; total mRNAs were obtained at 3 points on days 7, 14, and 21 and analyzed with DNA microarrays (Fig. 4a). Comparing *ROR2* KD cells with control cells, we identified 41 upregulated and 57 downregulated genes, 340 upregulated and 267 downregulated genes, 721 upregulated and 863 downregulated genes on days 7, 14, and 21, respectively (FC >|2| and FDR-adjusted *P-value* < 0.05; Supplementary Dataset 1). The top 10 genes with the highest differentially expressed fold-change in either direction are shown in Fig. 4b. At all differentiation time points, *NNAT* and *ZIC1* expression was strongly repressed in *ROR2* KD cells, and *PAX6* expression was the most upregulated on day 21. These differentially expressed genes were subjected to the ingenuity pathway analysis (IPA). The top five enriched canonical pathways of each time point are shown in Supplementary Fig. 3. The results showed a significant positive correlation between the synaptogenesis signaling pathway on days 14 (z-score = 2.353) and 21 (z-score = 2.887), indicating that *ROR2* KD facilitates NS/PC differentiation of hiPSCs even in the protocol using a medium other than the one used to identify the candidate marker genes.

The activation of WNT5A/ROR2 signaling in turn activates factors mediating the expression of the target gene associated with epithelial-mesenchymal transition (EMT)^16^. Ren et al. reported that the suppression of *ROR2* expression in epidermal carcinomas results in the downregulation of matrix metalloproteinase 2 (*MMP2*), which plays an important role in EMT^17^. Therefore, we investigated expression levels of EMT-related genes during the process of neuronal differentiation in control and *ROR2* KD cells; *MMP2* was significantly downregulated at days 14 and 21, and fibronectin 1 (*FN1*), a mesenchymal phenotypic marker, was significantly downregulated at day 21 in *ROR2* KD cells than in control cells (Supplementary Fig. 4). Our results suggest that *ROR2* functionally contributes to the EMT of hiPSCs during differentiation, which could cause enhanced NS/PC differentiation in *ROR2* KD cells.

### *ROR2* knockdown promotes differentiation into forebrain neurons

**Figure 5:**
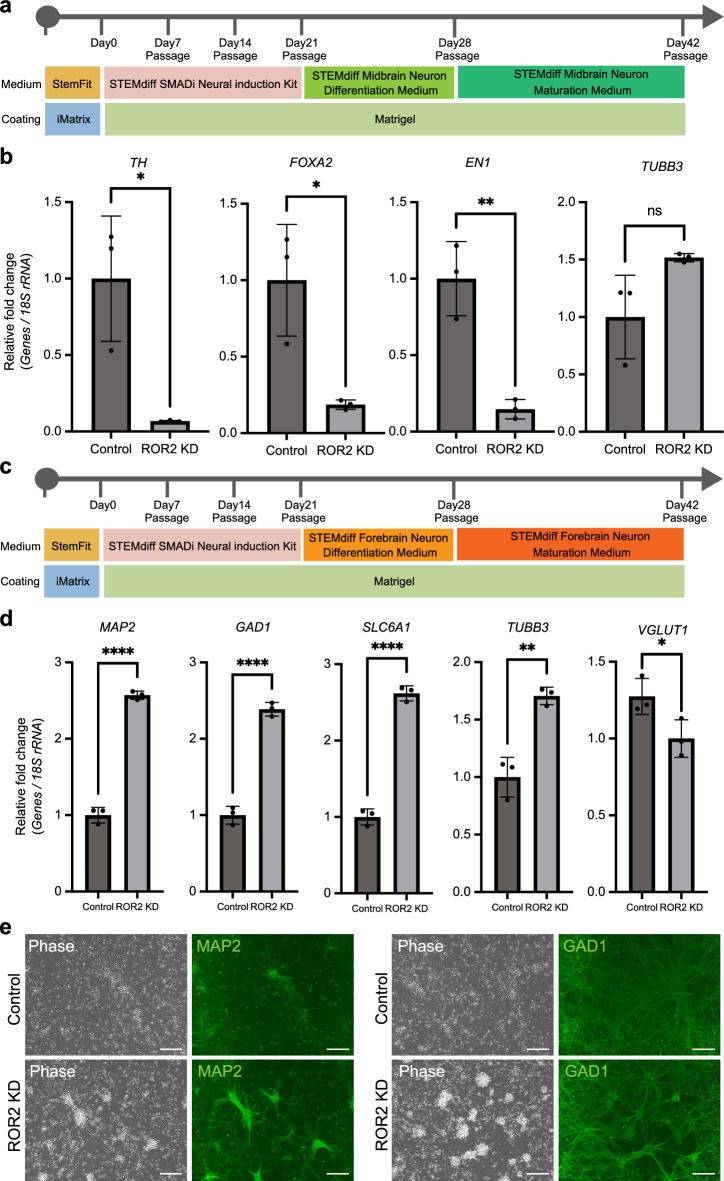
*ROR2* knockdown promotes differentiation into forebrain neurons. (**a**) Schematic of culture procedures for midbrain neuron differentiation. (**b**) qRT-PCR analysis of the mRNA levels of the midbrain neuron markers *TH*, *FOXA2*, *BN1,* and mature neuron marker *TUBB3*. Total RNA was isolated from R-2A *ROR2* KD cells and R-2A control shRNA cells that were differentiated into midbrain neurons (day 42, n = 3, biological replicates). (**c**) Schematic of culture procedures for forebrain neuron differentiation. (**d**) qRT-PCR analysis of the mRNA levels of the forebrain neuron markers *MAP2*, *GAD1*, *SLC6A1*, and *VGLUT1* and the mature neuron marker *TUBB3.* Total RNA was isolated from R-2A *ROR2* KD cells and R-2A control shRNA cells that were differentiated into forebrain neurons (day 42, n = 3, biological replicates). (**e**) Immunofluorescence staining of MAP2 (left) and GAD1 (right) in control (upper) and *ROR2* KD (lower) cell-derived forebrain neuron. Scale bars, 100 µm. *P < 0.05, **P < 0.01, ****P < 0.0001 (two-tailed unpaired t-test). Error bars represent mean ± SD.

We investigated the effect of *ROR2* KD on the hiPSC stage in neuronal differentiation after NS/PC. HiPSCs can differentiate into mature neurons such as dopaminergic neurons, GABAergic neurons, astrocytes, and oligodendrocytes. Treatment with Wnt5a, the ROR2 ligand, reportedly promotes differentiation into dopaminergic neurons^18^. We predicted that *ROR2* KD would inhibit the differentiation of hiPSCs into dopaminergic neurons. To test this hypothesis, we performed experiments where *ROR2* KD cells were differentiated into TH-positive dopaminergic neurons using a commercially available in vitro differentiation medium (Fig. 5a). Dopaminergic neuronal differentiation in hiPSCs was evaluated based on expression levels of representative markers of dopaminergic neurons using qRT-PCR. The *ROR2* KD cells exhibited altered marker profiles with reduced *TH*, *FOXA2,* and *EN1* expression (Fig. 5b). These results indicate that suppressing *ROR2* markedly inhibits dopaminergic neuron differentiation in hiPSCs, consistent with Wnt5a promoting this differentiation. Next, to confirm the function of *ROR2* in the differentiation of mature neurons, *ROR2* KD cells were differentiated into forebrain GABAergic neurons using a commercially available in vitro differentiation medium (Fig. 5c). *ROR2* KD cells after GABAergic neuronal differentiation showed significantly enhanced mRNA expression of GABAergic neuron markers *MAP2*, *GAD1,* and *SLC6A1.* Conversely, the expression of *VGLUT1*, a glutamatergic excitatory neuron marker, was decreased in *ROR2* KD cells (Fig. 5d). Moreover, immunofluorescence images indicated the upregulation of MAP2 and GAD1 protein expression in *ROR2* KD cells after inducing forebrain GABAergic neuron differentiation (Fig. 5e). To confirm our hypothesis that *ROR2* is a marker for GABAergic neuronal differentiation propensity, we employed two hiPSC lines showing distinct expression levels of *ROR2*: R-2A and R-1A cell lines were used as high and low *ROR2*-expressing cell lines, respectively. As expected from the *ROR2* expression levels in hiPSC lines, GABAergic neurons derived from R-1A cells exhibited a higher expression of GABAergic neuron marker genes, compared with R-2A cells (Supplementary Fig. 5). These results suggest that *ROR2* is a predictive marker for the differentiation of hiPSCs into forebrain GABAergic neurons. Thus, the evaluation of *ROR2* expression levels would allow the selection of hiPSC lines with a high propensity for NS/PC and forebrain GABAergic neuron differentiation.

## Discussion

In this study, we expressed the differentiation efficiency of each hiPSC line as PC1 of differentiation markers (*PAX6*, *SOX1*, and *NES*) and extracted differentiation predictive markers by performing Spearman's rank correlation analysis with gene expression in the undifferentiated state. Furthermore, we selected superior differentiation-predictive markers by performing independent correlation analyses using two different neural differentiation methods and extracting genes with a common correlation in the extracted gene set. Loss-of-function studies have shown that the *ROR2* identified in this study is a versatile marker regulating the differentiation of hiPSCs into NS/PCs.

Using the same method, we previously identified *SALL3*, a marker of three-germ layer differentiation^10^, and *CXCL4*, a marker of cardiac differentiation^11^, suggesting that our approach facilitates the identification of predictive markers regulating differentiation. However, the utility of these genes as markers may still depend on the differentiation protocols. In contrast, the *ROR2* KD cells showed enhanced neuronal differentiation even when the differentiation method was changed, indicating that *ROR2* plays a critical role in NS/PC differentiation and can serve as a versatile marker regardless of the differentiation protocol.

The WNT signal is critical for hiPSC differentiation and essential in neural differentiation. Its complex effects vary depending on the timing of the differentiation process^19,20^. Kirkeby et al. have reported that WNT pathway activation by small molecules, including CHIR99021 (CHIR), exerts a precise dose-dependent effect in patterning the NS/PCs to forebrain neurons (including glutamatergic excitatory neuron and GABAergic neurons), and midbrain neurons (including dopaminergic neuron). At a low concentration of CHIR, the NS/PCs become progenitors of forebrain neurons. With increasing concentrations, the NS/PCs are fated to become midbrain neurons^21^. Moreover, Wnt5a, which activates the non-canonical Wnt signaling, promotes the dopaminergic neuronal differentiation of neural stem cells^18^. Thus, the suppression of WNT5A/ROR2 signaling by *ROR2* KD could accounts for two pathways simultaneously: suppression of differentiation into midbrain neurons and promotion of differentiation into forebrain neurons. Therefore, it would be beneficial to select hiPSCs with low *ROR2* expression as raw material for efficiently producing NS/PCs or GABAergic neurons from hiPSCs. Furthermore, activation of *ROR2* in NS/PCs would be useful for the efficient production of dopaminergic neurons from NS/PCs.

The activation of WNT5A/ROR2 signaling results in the activation of factors mediating the expression of the target gene associated with EMT^16^. During embryonic development, epiblastic cells of the primitive streak undergo EMT for internal movement and generate two inner layers, the mesoderm and endoderm, while differentiation of the remaining epiblast generates the ectoderm^22^. Suppressing EMT activation through WNT5A/ROR2 signaling by *ROR2* KD may suppress hiPSC differentiation into the mesoderm/endoderm and consequently activate NS/PC differentiation through the ectoderm during the induction of NS/PC differentiation. We examined expression levels of EMT-related genes using microarray data and found that *MMP2* was significantly downregulated in *ROR2* KD cells on days 14 and 21 and *FN1* on day 21. Our results are consistent with reports that the suppression of *ROR2* expression in epidermal carcinomas results in the downregulation of *MMP2*^17^. In addition, the EMT is essential for neural crest development^23^. We compared gene-expression profiles during the differentiation of hiPSCs into late-stage NS/PCs between *ROR2* KD cells and control cells and found that *ZIC1* expression, which is vital for NC differentiation^24^, was markedly reduced in *ROR2* KD cells than in controls between days 7 and 21 of NS/PC differentiation. EMT inactivation would suppress differentiation to the neural crest, which could cause enhanced NS/PC differentiation in *ROR2* KD cells. *MMP2* is known to promote the migration of neural crest cells in embryo^25^, paralleling the behavior of *ZIC1* expression observed in our results.

In conclusion, our proposed cell line selection method can overcome differences in differentiation potential among disparate hiPSC lines. Our study proposes a method for identifying markers in the pluripotent state used in predicting NS/PC differentiation behavior in hiPSC lines using rank correlation. This approach could be used to identify differentiation prediction markers for other hiPSCs. We believe that this study is a crucial assessment of the quality of hiPSC raw materials and considers standardizing hiPSC lines used for cell therapy.

## Methods

### Cell culture

Commercially available hiPSC lines were used in this study (Supplementary Table 1). HiPSC lines were obtained from RIKEN Cell Bank (201B7, 253G1, 409B2, HiPS-RIKEN-1A, HiPS-RIKEN-2A, and HiPS-RIKEN-12A), American Type Culture Collection (ATCC-DYR0110 hiPSC and ATCC-HYR01103 hiPSC), JCRB Cell Bank (Tic), and System Biosciences (human mc-iPS). HiPSCs were screened for mycoplasma contamination and hiPSCs used in this study were mycoplasma-free. Undifferentiated hiPSCs were maintained on an iMatrix-511 (Nippi) in StemFit AK02 medium (Ajinomoto). All cells were cultured at 37 °C in a humidified atmosphere containing 5% CO_2_ and 95% air.

### Neural stem/progenitor cell differentiation of hiPSCs

Differentiation of hiPSCs into NS/PCs was induced, as previously reported, with a few modifications. For adhesive differentiation, hiPSCs were detached through incubation with StemPro Accutase (Thermo Fisher Scientific) containing 10 μM Y-27632 for 10 min and seeded onto 24-well cell culture plates (BD Biosciences) coated with iMatrix at a density of 25,000 cells/cm^2^ for 2–3 days before NS/PC induction. Confluent hiPSCs were treated with 10 μM of the ALK inhibitor SB431542 (Stemgent) and 500 ng/mL of Noggin (R&D systems) in DMEM/F12 medium containing 20% KSR. The medium was replaced on days 1 and 2. On day 6 of differentiation, SB431542 was withdrawn, and increasing amounts of N2 media (25%, 50%, and 75%) were added to the knockout serum replacement medium every 2 days while maintaining 500 ng/mL of Noggin. For suspension differentiation, hiPSCs were treated with 10 μM Y-27632 for 1 h at 37 °C and dissociated with StemPro Accutase (Thermo Fisher Scientific) containing 10 μM Y-27632 for 10 min to generate single-cell suspensions and suspended in B27N2-based medium [DMEM/F12 with 15 mM HEPES, 5% B27, and 5% N2 supplements (Life Technologies), 10 μM SB431542, 2 μM Dorsomorphin (Fujifilm), and 10 ng/mL bFGF (R&D systems)]. The completely dissociated cells were seeded into ultralow attachment 96-well plates (PrimeSurface^®^ 96-well, Sumitomo Bakelite) at 9,000 cells/well, centrifuged at 700 g for 3 min (quick aggregation). The medium was changed daily for up to 10 days; for the first 3 days, 10 µM of Y-27632 was added. Total RNA was obtained from 40 wells of neuro spheres per sample. For microarray analysis, hiPSCs were differentiated into NS/PCs using a STEMdiff SMADi Neural Induction Kit (Stem Cell Technologies) according to the manufacturer’s instructions. Briefly, hiPSCs were maintained on an iMatrix-coated plate in StemFitAK02 media (Ajinomoto) before NS/PC induction. Cells were harvested using Accutase (Thermo Fisher Scientific); 2 × 10^6^ cells were transferred to a Matrigel-coated 6-well plate in STEMdiff Neural Induction Medium + SMADi (Stem Cell Technologies) supplemented with 10 μM Y-27632. The medium was replenished daily with warmed (37 °C) STEMdiff Neural Induction Medium + SMADi until the culture was terminated. Cells were passaged every 7 days, and RNA was extracted from cells harvested at passages (days 7, 14, and 21).

### qRT-PCR and PCA

Total RNA was isolated from hiPSCs or differentiated cells using the RNeasy Mini Kit (Qiagen) and treated with DNase I according to the manufacturer’s instructions. qRT-PCR was performed using a QuantiTect Probe One-Step RT-PCR Kit (Qiagen) on a STEPONEPLUS Real-Time PCR System (Applied Biosystems). The expression levels of target genes were normalized to those of the *GAPDH* transcript or 18S rRNA, which were quantified using TaqMan human Glyceraldehyde-3-phosphate dehydrogenase (GAPDH) control reagents (Applied Biosystems) or eukaryotic 18S rRNA endogenous controls (Applied Biosystems), respectively. The probes and primers were obtained from Sigma-Aldrich. The used primer and probe sequences are listed in Supplementary Table 2. PCA was performed using SYSTAT 13 software (Systat Software Inc.) after data standardization (z-scoring) for each NS/PC marker gene.

### Rank correlation analysis

To identify microarray probe sets related to the differentiation of hiPSCs into NS/PC, correlations between the intensity value rank of the filtered probe sets and the PC1 rank in the 10 hiPSC lines were determined by calculating Spearman’s rank correlation coefficients (*r*_s_), as described in a previous study^26^. Probe sets exhibiting statistically significant correlations (*P* < 0.01) were selected. When n = 10 data points, the observed value of *r*_s_ should exceed 0.794 (positively correlated) or less than –0.794 (negatively correlated) to be considered statistically significant (*P* < 0.01).

### Lentivirus-derived RNAi and generation of ROR2 KD cell lines

*ROR2* KD cells were generated by infecting R-2A cells with MISSION Lentiviral Transduction Particle expressing *ROR2*-targeted shRNAs (#1: TRCN0000199888, #2: TRCN0000001492) or MISSION^®^pLKO.1-puro Control Non-Mammalian shRNA Control Transduction Articles (Sigma, SHC002V), according to the manufacturer’s instructions. Media containing viruses were collected 48 h after transfection, and the cells were transduced with the viruses using 8 µg/mL polybrene (Sigma-Aldrich) for 24 h. The cells were selected using 2 µg/mL puromycin (Gibco) for 48 h.

### Western blotting analysis

The cell lysates were used for western blotting analysis. Proteins were separated using sodium dodecyl sulfate–polyacrylamide gel electrophoresis, transferred to PVDF membranes (Bio-Rad), and blocked for 60 min in Blocking One (Nacalai tesque). Primary antibody dilutions were prepared in Can Get Signal immunoreaction enhancer solution (TOYOBO) as follows: anti-ROR2 antibody (AF2064; R&D Systems) 1:1000, anti-β-actin antibody (A5441; Sigma-Aldrich) 1:2000. Membranes were incubated with HRP-conjugated anti-mouse IgG (Invitrogen) or anti-goat IgG (Invitrogen). Proteins were visualized using ECL Prime Western Blotting Detection Reagent (GE Healthcare) and the ChemiDoc Touch Imaging System (Bio-Rad).

### Immunofluorescence staining

HiPSC-derived NS/PC or forebrain neuron was fixed in 4% paraformaldehyde in PBS (Nacalai) for 20 min at 25 °C. After washing with PBS, the cells were permeabilized with 0.2% Triton-X100 (Merk) in PBS for 15 min and blocked with Blocking One (Nacalai) for 30 min. The samples were incubated for 1 h with primary antibodies (anti-PAX6 antibody [PRB-278P-100, BioLegend], anti-MAP2 antibody [MAB8304, R&D systems], and anti-GAD1 antibody [AF2086, BioLegend]). Indirect immunostaining was performed with the secondary antibody (anti-rabbit IgG/Alexa Fluor 555 [A27039, Thermo Fisher Scientific], anti-goat IgG/Alexa Fluor 488 [A32814, Thermo Fisher Scientific], and anti-mouse IgG/Alexa Fluor 488 [A28175, Thermo Fisher Scientific]) for 1 h and examined under a BZ-X810 fluorescence microscope (Keyence).

### Generation of a *ROR2*-overexpressing cell line

*ROR2* overexpression cells were generated by infecting 253G1 cells with lentiviral particles expressing *ROR2*. Briefly, the nucleotide sequence of the human *ROR2* open reading frame (NM_004560) was de novo synthesized (Eurofins Genomics) and cloned into the pLVSIN-EF1α puromycin vector (Takara Clontech). Lentivirus packaging and virus infection were performed as described above.

### Microarray (Clariom D Assay)

Total RNA was extracted from hiPSC-derived NS/PC cells using an RNeasy Mini Kit (QIAGEN) according to the manufacturer’s instructions. Total RNA (100 ng per sample) was used as the input for the Clariom D Assay (Thermo Fisher Scientific). Target preparation was performed using a Gene Chip™ WT PLUS Reagent Kit (Thermo Fisher Scientific) according to the manufacturer’s instructions. Hybridization was performed in a Gene Chip Hybridization Oven 645 for 16 h at 45 °C. Gene chips were scanned using a GeneChip Scanner 3000. Array quality control was performed using Transcriptome Analysis Console software (version 4.0.2.15). The National Center for Biotechnology Information Gene Expression Omnibus (NCBI GEO) accession number for the microarray data is GSE233228.

### Mature neuron differentiation

Differentiation of hiPSCs into mature nerves was performed according to the manufacturer’s instructions using the STEMdiff Forebrain Neuron Differentiation Kit (#08600, STEMCELL Technologies) for forebrain-type nerves and the STEMdiff Midbrain Neuron Differentiation Kit (#100-0038, STEMCELL Technologies) for midbrain nerves. Using the STEMdiff SMADi Neural Induction Kit (Stem Cell Technologies) monolayer culture protocol described above, hiPSCs were differentiated into NS/PC, and mature neural differentiation was induced.

For midbrain neuron differentiation, hiPSC-derived NS/PCs (day 21, passage 3) were detached using Accutase and seeded into PLO (Sigma)-and laminin (Sigma)-coated 12-well plate at a density of 1.25 × 10^5^ cells/cm^2^ culture in STEMdiff Neural Induction Medium + SMADi medium for 24 h. The complete medium was replaced daily for 6 days with STEMdiff Midbrain Neuron Differentiation Medium. The midbrain neural precursors (day 7) were detached using ACCUTASE and seeded into PLO-and Laminin-coated 12-well plate at a density of 5 × 10^4^ cells/cm^2^ in STEMdiff Midbrain Neuron Maturation medium with a half-medium change every 2–3 days for 14 days.

For forebrain-type neuron differentiation, hiPSC-derived NS/PCs (day 21, passage 3) were detached using Accutase and then seeded into PLO-and Laminin-coated 12-well plate at a density of 1.25 × 10^5^ cells/cm^2^ culture in STEMdiff Neural Induction Medium + SMADi medium for 24 h. The full medium was replaced daily for 6 days with STEMdiff Forebrain Neuron Differentiation medium. The forebrain neural precursors (day 7) were detached using Accutase and seeded into PLO- and Laminin-coated 12-well plate at a density of 5 × 10^4^ cells/cm^2^ in STEMdiff Forebrain Neuron Maturation media with a half-medium change every 2–3 days for 14 days.

### Statistical analysis

Statistical analyses were performed using Prism 9 software (version 9.5.1; GraphPad Software Inc.). Data are presented as mean ± standard deviation (SD). For comparison between two groups the t-test was applied; in cases where another statistic test was applied, it is mentioned accordingly. Statistical significance was set at *P* < 0.05.

### Supplementary Information

Supplementary Information 1.

Supplementary Information 2.

## Data Availability

Data supporting the findings of this study are available in the article and supplementary information. The analyzed microarray data have been deposited in NCBI’s Gene Expression Omnibus (GEO) and are accessible through the GEO Series accession number GSE233228. All remaining raw data are accessible from the corresponding author upon request. The source data are provided in this study.
