# Supplementary material for: ROR2 expression predicts human induced pluripotent stem cell differentiation into neural stem/progenitor cells and GABAergic neurons
Source: Sci Rep. 2024 Jan 6;14:690. doi: 10.1038/s41598-023-51082-4 (PMC10771438; doi:10.1038/s41598-023-51082-4)
Supplement: Supplementary file 2 — Supplementary Information 2. [file 41598_2023_51082_MOESM2_ESM.pdf]

Supplementary Information

ROR2 expression predicts human induced pluripotent stem cell differentiation into neural stem/progenitor cells and GABAergic neurons

Takuya Kuroda[1], Satoshi Yasuda[1,2,3], Satoko Matsuyama[1,4], Takumi Miura[1,2,5], Rumi Sawada[1], Akifumi Matsuyama[4], Yumiko Yamamoto[6], Masaki Suimye Morioka[6], Hideya Kawaji[6,7], Takeya Kasukawa[6], Masayoshi Itoh[6], Hidenori Akutsu[5], Jun Kawai[2,6], and Yoji Sato[1,2,8,9*]

1. Division of Cell-Based Therapeutic Products, National Institute of Health Sciences, Kanagawa, Japan
2. Life Science Technology Project, Kanagawa Institute of Industrial Science and Technology, Kanagawa, Japan
3. Department of Quality Assurance Science for Pharmaceuticals, Graduate School of Pharmaceutical Sciences, Nagoya City University, Aichi, Japan
4. Center for Reverse TR, Osaka Habikino Medical Center, Osaka Prefectural Hospital Organization, Osaka, Japan
5. Center for Regenerative Medicine, National Center for Child Health and Development, Tokyo, Japan
6. RIKEN Center for Integrative Medical Sciences, Kanagawa, Japan
7. Research Center for Genome & Medical Sciences, Tokyo Metropolitan Institute of Medical Science, Tokyo, Japan
8. Division of Drugs, National Institute of Health Sciences, Kanagawa, Japan

9. Department of Cellular and Gene Therapy Products, Graduate School of Pharmaceutical Sciences, Osaka University, Osaka, Japan

*Corresponding Author: Yoji Sato, Ph.D., Division of Drugs, National Institute of Health Sciences, 3-25-26 Tonomachi, Kawasaki-ku, Kawasaki-shi, Kanagawa, 210-9501, Japan

Phone: +81-44-270-6505. Fax: +81-44-270-6506. E-mail: yoji@nihs.go.jp

Supplementary Figure 1

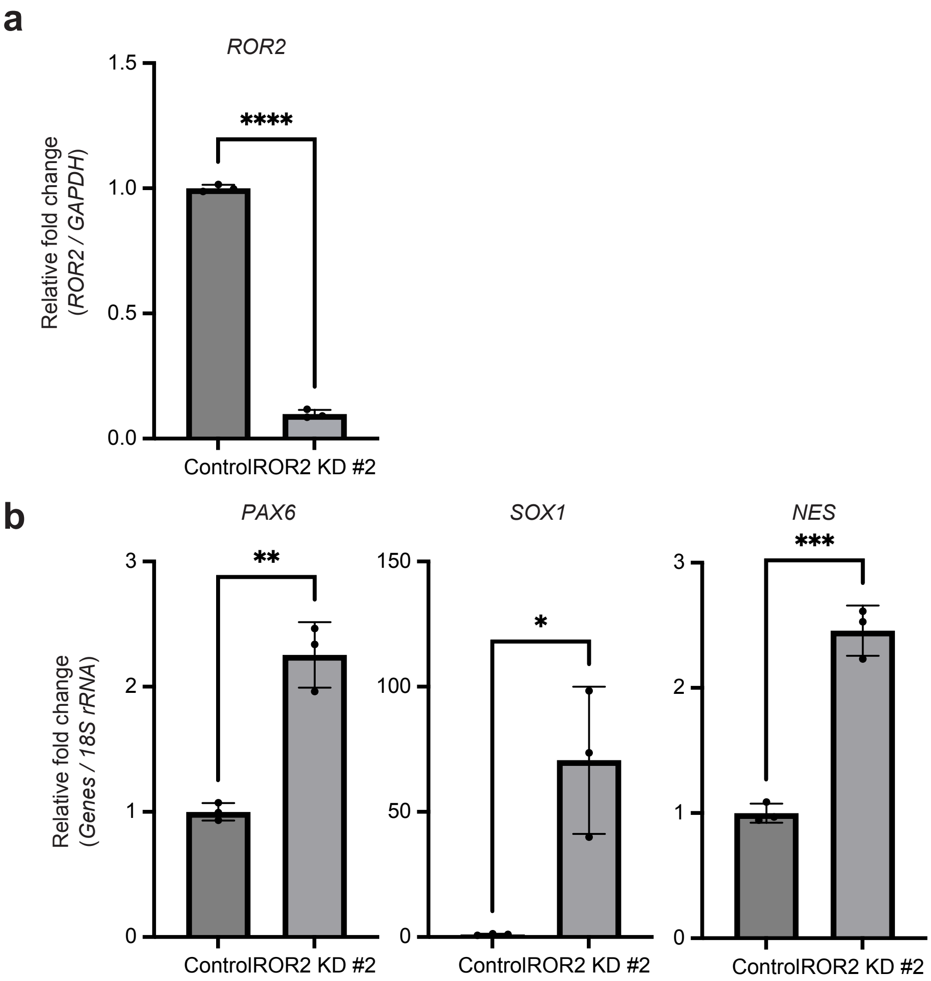

Supplementary Figure 1. NS/PC differentiation of R-2A *ROR2* KD (shRNA clone-#2)

(A) *ROR2* knockdown was confirmed by qRT-PCR analysis (n = 3, biological replicates). (B) R-2A *ROR2* KD-#2 cells were differentiated to NS/PCs with suspension method. qRT-PCR analysis of NS/PC marker genes in NS/PCs derived from *R0R2* KD-#2 cells and control shRNA cells (n = 3, biological replicates). *$P < 0.05$, **$P < 0.01$, ***$P < 0.005$,****$P < 0.0001$ (two-tailed unpaired t-test). Error bars represent mean ± SD.

Supplementary Figure 2

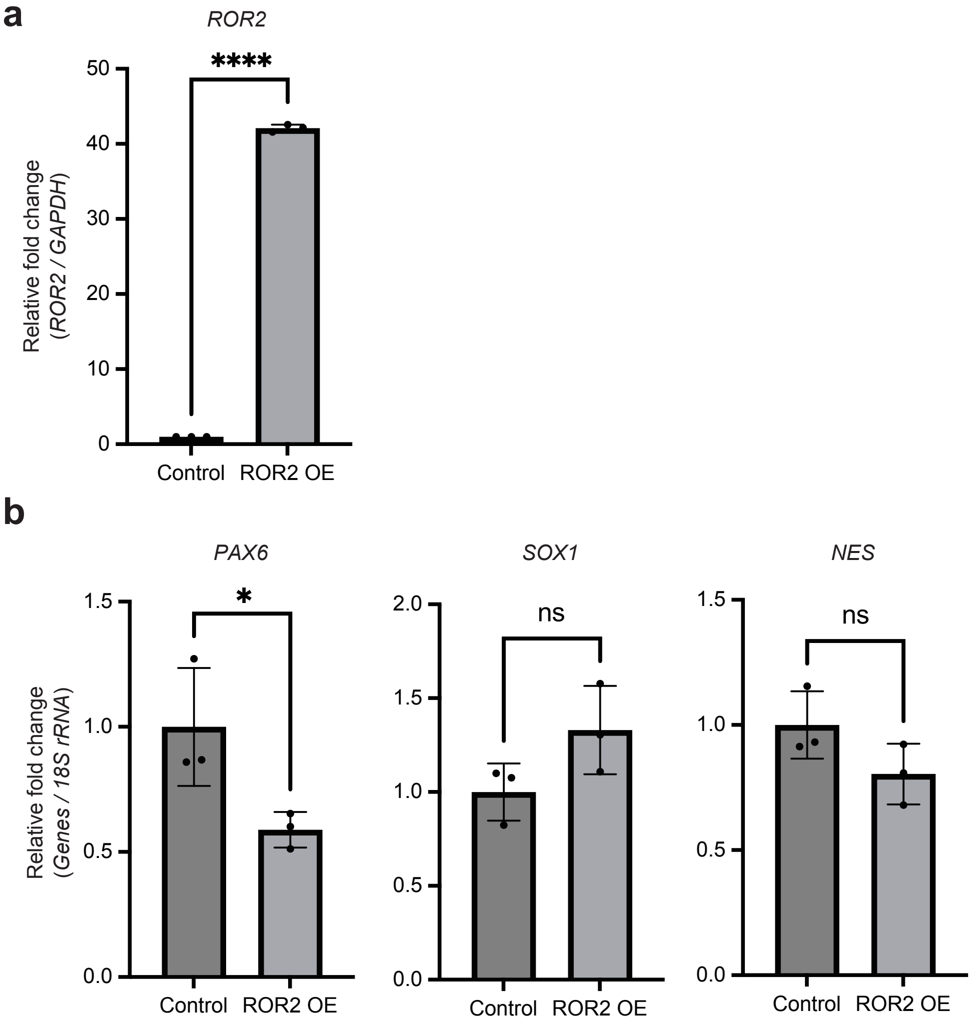

Supplementary Figure 2. NS/PCs differentiation of 253G1 *ROR2*-overexpression cells

(a) *ROR2* mRNA levels in undifferentiated 253G1 *ROR2* overexpressing cells (*ROR2* OE). (n = 3, biological replicates). **(b)** 253G1 *ROR2* overexpressing cells were differentiated to NS/PCs with adhesion method. qRT-PCR analysis of NS/PCs marker genes in NS/PCs derived from 253G1 *ROR2* overexpressing cells cells and control cells (n = 3, biological replicates). *$P < 0.05$, ****$P < 0.0001$ (two-tailed unpaired t-test). Error bars represent mean ± SD.

Supplementary Figure 3

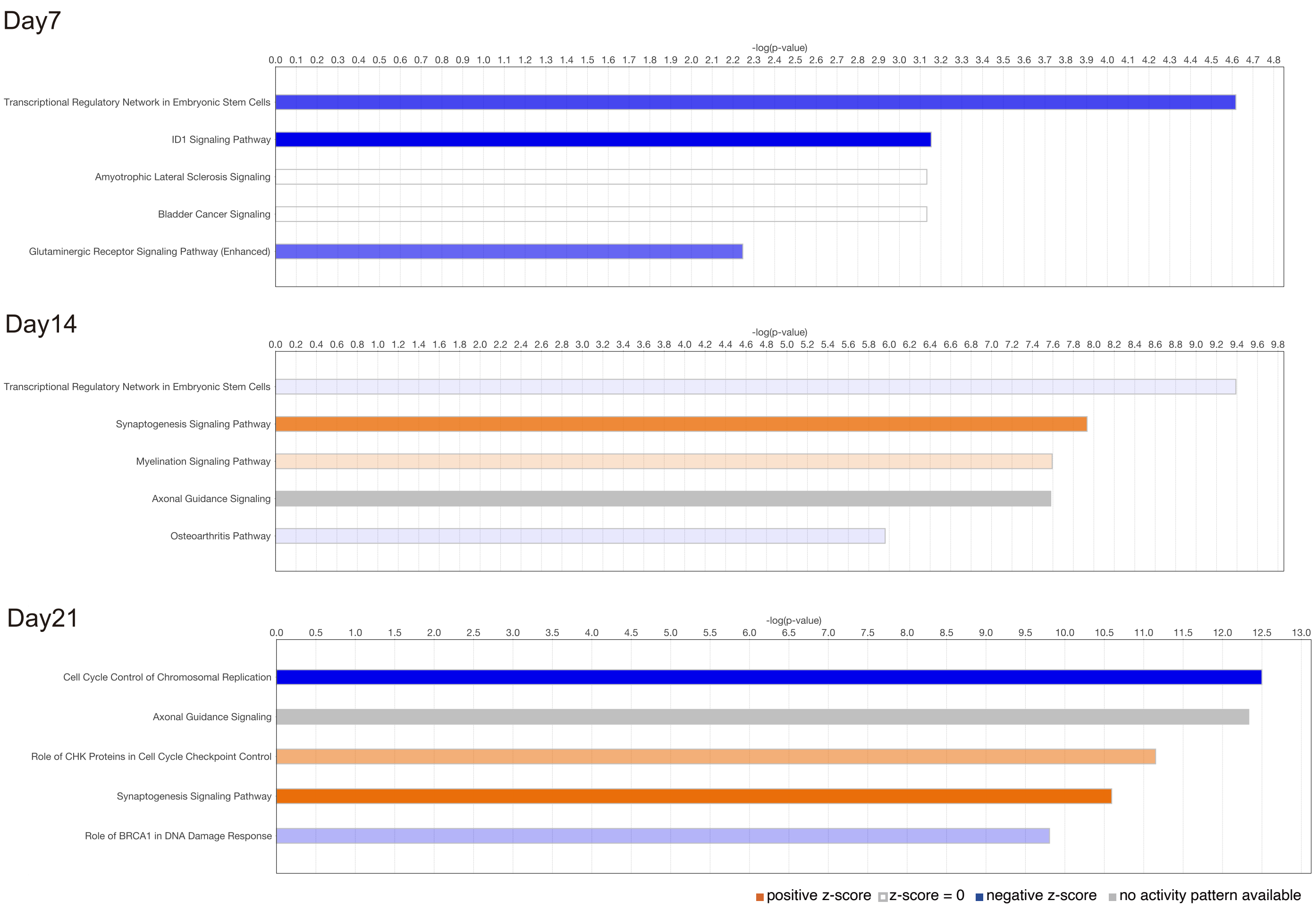

Supplementary Figure 3. Pathway analysis

Differentially expressed genes were subjected to IPA. The top five canonical pathways enriched at each time point are shown.

Supplementary Figure 4

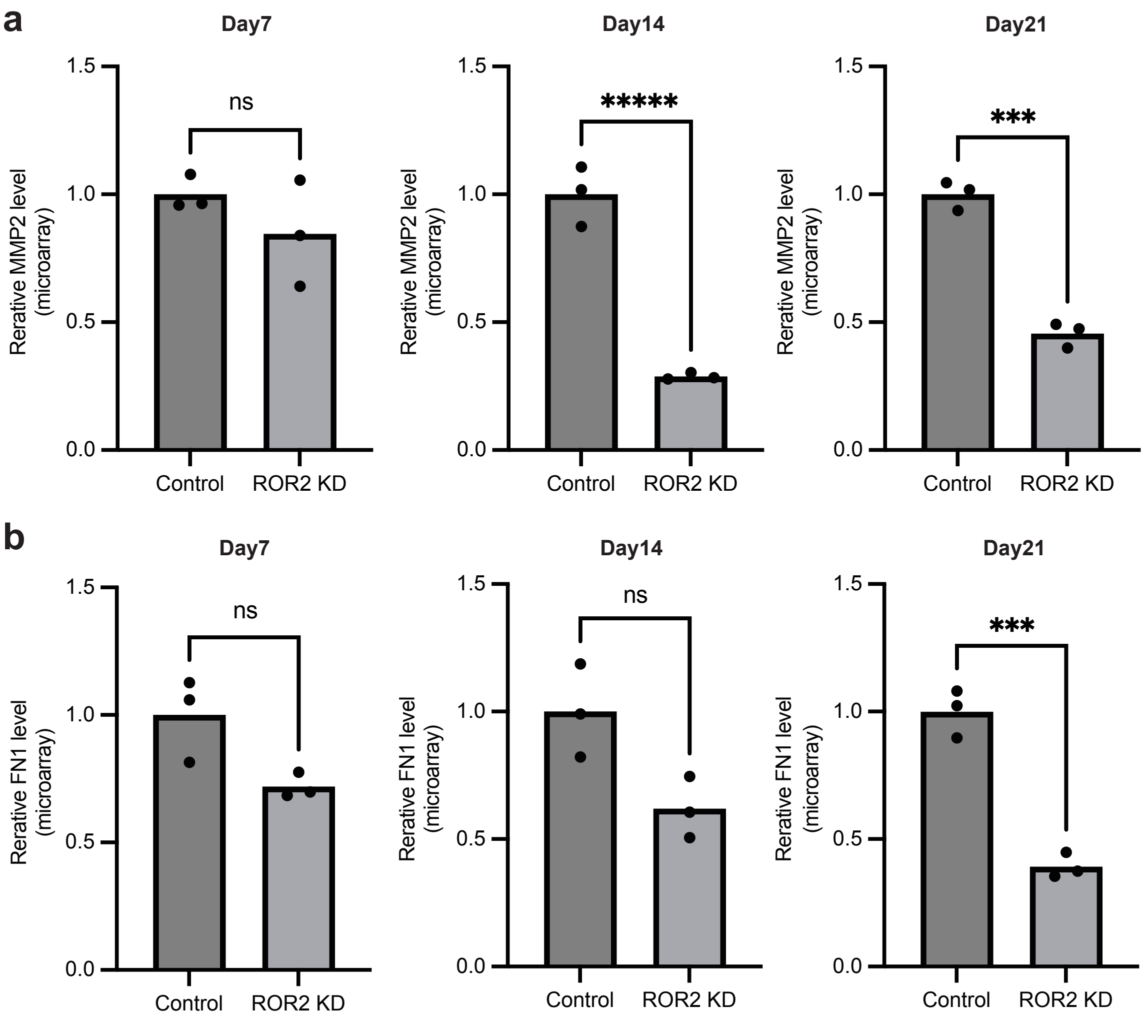

Supplementary Figure 4. EMT-related genes affected by *ROR2* knockdown in NS/PC differentiation

Comparison of *MMP2* and *FN1* expression between control and *ROR2* KD cells during neuronal differentiation process with microarray expression analysis.(n = 3, biological replicates). ***$P < 0.001$, *****$P < 0.000001$ (FDR-adjusted p-value). FDR-adjusted p-values were calculated with Transcriptome Analysis Console software (Thermo Fisher Scientific, version 4.0.2.15)

Supplementary Figure 5

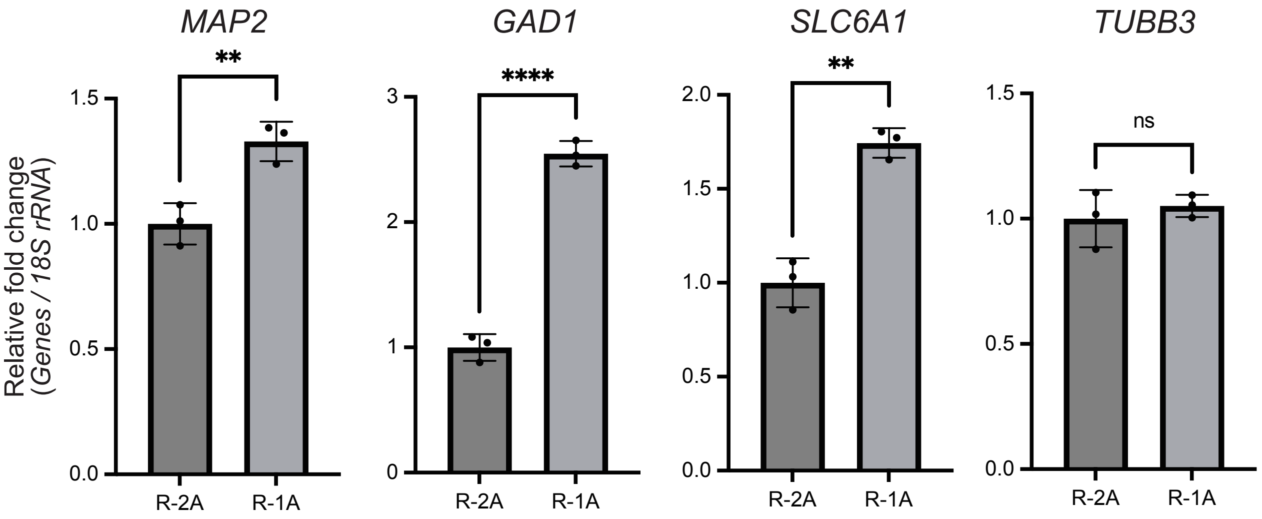

Supplementary Figure 5. Forebrain neuron differentiation of R-2A cell and R-1A cell.

qRT-PCR analysis of the mRNA levels of the forebrain neuron markers *MAP2*, *GAD1*, *SLC6A1*, and mature neuron marker *TUBB3*. Total RNA was isolated from R-2A cells and R-1A cells that were differentiated into forebrain neurons (day42, n = 3, biological replicates). $^{**}P < 0.01$, $^{****}P < 0.0001$ (two-tailed unpaired t-test). Error bars represent mean ± SD.

Supplementary Table 1. Information about the hiPSC lines

Cell Line (abbreviation)	Trans gene	Method of reprograming	Cell source	Donor Age	Donor Sex	Passage# Microarray (in-house passage#)	Reference
201B7	OCT3/4, SOX2, KLF4, c-MYC	Retrovirus	Human dermal fibroblasts	36	Female	27	Takahashi K, et al. Cell. 2007 Nov 30;131(5):861-72.
253G1	OCT3/4, SOX2, KLF4	Retrovirus	Human dermal fibroblasts	36	Female	32	Nakagawa M, et al. Nat Biotechnol. 2008 Jan;26(1):101-6. Epub 2007 Nov 30.
409B2	OCT3/4, SOX2, KLF4, L-MYC, LIN28, p53-shRNA	Episomal vector	Human dermal fibroblasts	36	Female	42	Okita K, et al. Nat Methods. 2011 May;8(5):409-12.
mc-iPS	OCT3/4, SOX2, KLF4, c-MYC	Plasmid	Human adipose stem cells	40-65	Female	(14)	Jia F, et al. Nat Methods. 2010 Mar;7(3):197-9.
Tic	OCT3/4, SOX2, KLF4, c-MYC	Retrovirus	Human fetus lung cells (MRC-5)	–	Male	50	Fujioka T, et al. Hum Cell. 2010 Aug;23(3):113-8
ATCC-DYR0100hiPSc (DYR0100)	OCT3/4, SOX2, KLF4, c-MYC	Retrovirus	Neonatal dermal fibroblasts	Newborn	Male	(5)	
ATCC-HYR0103iPSc	OCT3/4, SOX2, KLF4, c-MYC	Retrovirus	Hepatic fibroblast	31	Male	(5)	

(HYR0103)							
HiPS-RIKEN-1A (R-1A)	OCT3/4, SOX2, KLF4, c-MYC	Retrovirus	Umbilical cord-derived fibroblasts	–	Female	21	Fujioka T, et al. Hum Cell. 2010 Aug;23(3):113-8
HiPS-RIKEN-2A (R-2A)	OCT3/4, SOX2, KLF4, c-MYC	Retrovirus	Umbilical cord-derived fibroblasts	–	Male	21	Fujioka T, et al. Hum Cell. 2010 Aug;23(3):113-8
HiPS-RIKEN-12A (R-12A)	OCT3/4, SOX2, KLF4	Retrovirus	Umbilical cord-derived fibroblasts	–	Male	16	Fujioka T, et al. Hum Cell. 2010 Aug;23(3):113-8

Supplementary Table 2. Probe and primer sequences.

Gene	FAM-TAMRA Probe sequences (5’ →3’)	Forward primer sequences (5’→3’)	Reverse primer sequences (5’→3’)
LIN28A	CGCATGGGGTTCGGCTTCCTGTCC	CACGGTGCGGGCATCTG	CCTTCCATGTGCAGCTTACTC
OCT3/4	CGGACCACATCCTTCTCGAGCCCAAGC	GAAACCCACACTGCAGCAGA	TCGCTTGCCCTTCTGGCG
PAX6	CCAGCCAGACCTCCTCATACTCCTGCAT	GGCAAATAACCTGCCTATGCAAC	ACTCCGCCCATTCACCGAA
SOX1	AGAAAACGCTTTCCGCTTCCTCCGTAGG	GACTGAACTTCGGTGTTTTCTTGA	GCCTCTCGCCTCGTTTTGAC
NES	TCTGTAGGCCCTGTTTCTCCTGCTCCA	GGCAGCGTTGGAACAGAGG	CCTTCCAGGACCTGAGCGA
ROR2	CCGAACGACCCTTTAGGACCCCTTGATGG	GTGTCCCGGACTTCAGGTGA	GGCTCCAGAAAATTCAGAAAGTAACC
TH	AGGACAAGCTCAGGAGCTATGCCTCACG	CGTGTCTGAGAGCTTCAGTGAC	ACGGGTCGAACTTCACGGA
FOXA2	AGGGCTACTCCTCCGTGAGCAACATGA	CCTGCATTCTCTGATGACAAGTTC	GCTTCACAAGGTTAATGAGAAACTC
EN1	ACTGCACACGTTATTCGGATCGTCCATCCT	TCGCAGCAGCCTCTCGTAT	CCTTCTCGTTCTTCTTCTTCTTCAG
TUBB3	CTGGAGCGGATCAGCGTCTACTACAACG	CTACGTGGGCGACTCGGA	CCCCACTCTGACCAAAGATGAAA
MAP2	CCACCTGCTGCTTCCTCCACTGTGAC	ACAGCTAATCTGCCTCCTTCTC	GTCCTTTGCCTGTTTAAATACACTG
GAD1	AGGCAATCCTCCAAGAACCTGCTTTCCTGT	CTGGAAGAGAAGAGTCGCCTTG	GCCGGAAGCAGATCTCTAGC
SLC6A1	ACAACTCCTTCACCACGACACTGCCG	CTCCTGGGCCATTTACTACCTG	GGAGAAGCAGCGGTCTGTG
GFAP	CGGCTGCGGCTCGATCAACTCACC	CTACCAGGCTGAGCTGCGA	TCATCCTGGAGCTTCTGCCT
VGLUT1	AGTACGTGTTCCTAATTGCCTCCCTGGTGC	CCATGACTAAGCACAAGACTCGG	TCGCTCATCTCCTCAGGCTC

Supplementary Dataset 1. List of genes differentially expressed during NS/PC differentiation between *ROR2* KD cells and control cells

ROR2 KD cells and control cells were compared for gene expression profiles during differentiation of hiPSCs into NS/PC to explore possible molecular mechanisms underlying the negative effects of *ROR2* on NS/PC differentiation. By comparing *ROR2* KD cells with control cells, we identified 41 upregulated and 57 downregulated genes, 340 upregulated and 267 downregulated genes, 721 upregulated and 863 downregulated genes on days 7, 14, and 21, respectively (FC > |2| and FDR-adjusted *p-value* < 0.05). For each of these genes, the gene symbol, fold change, signal values, and FDR p-val are shown.
